# 

**Published:** 1868-03-15

**Authors:** 


Vol. XXV. Number 6.

### THE CHICAGO MEDICAL JOURNAL.

*PUBLISHED SEMI-MONTHLY.*

EDITED BY

J. Adams Allen, M.D., LL.D.

MARCH 15, 1868.

CHICAGO:

Church, Goodman and Donnelley, Steam Book and Job Printers,

108 and 110 Dearborn Street.

